# 5′UTR Variants of Ribosomal Protein S19 Transcript Determine Translational Efficiency: Implications for Diamond-Blackfan Anemia and Tissue Variability

**DOI:** 10.1371/journal.pone.0017672

**Published:** 2011-03-11

**Authors:** Jitendra Badhai, Jens Schuster, Olof Gidlöf, Niklas Dahl

**Affiliations:** Department of Immunology, Genetics and Pathology, Science for Life Laboratory and Rudbeck Laboratory, Uppsala University, Uppsala, Sweden; University of Louisville, United States of America

## Abstract

**Background:**

Diamond-Blackfan anemia (DBA) is a lineage specific and congenital erythroblastopenia. The disease is associated with mutations in genes encoding ribosomal proteins resulting in perturbed ribosomal subunit biosynthesis. The *RPS19* gene is mutated in approximately 25% of DBA patients and a variety of coding mutations have been described, all presumably leading to haploinsufficiency. A subset of patients carries rare polymorphic sequence variants within the 5′untranslated region (5′UTR) of *RPS19*. The functional significance of these variants remains unclear.

**Methodology/Principal Findings:**

We analyzed the distribution of transcriptional start sites (TSS) for *RPS19* mRNAs in testis and K562 cells. Twenty-nine novel *RPS19* transcripts were identified with different 5′UTR length. Quantification of expressed w.t. 5′UTR variants revealed that a short 5′UTR correlates with high levels of RPS19. The total levels of *RPS19* transcripts showed a broad variation between tissues. We also expressed three polymorphic *RPS19* 5′UTR variants identified in DBA patients. The sequence variants include two insertions (c.-147_-146insGCCA and c.-147_-146insAGCC) and one deletion (c.-144_-141delTTTC). The three 5′UTR polymorphisms are associated with a 20–30% reduction in RPS19 protein levels when compared to the wild-type (w.t.) 5′UTR of corresponding length.

**Conclusions:**

The *RPS19* gene uses a broad range of TSS and a short 5′UTR is associated with increased levels of RPS19. Comparisons between tissues showed a broad variation in the total amount of *RPS19* mRNA and in the distribution of TSS used. Furthermore, our results indicate that rare polymorphic 5′UTR variants reduce RPS19 protein levels with implications for Diamond-Blackfan anemia.

## Introduction

Diamond-Blackfan anemia (DBA; OMIM #205900) is a rare congenital bone marrow failure characterized by decreased numbers or absence of erythroid precursor cells [1]. Approximately 50–60% of DBA patients carry a mutation in one of nine ribosomal protein (RP) genes of which *RPS19* mutations account for 25% [2]. A series of >100 coding mutations in the *RPS19* gene have been identified ranging from deletions and insertions of various sizes, single base substitutions resulting in both non-sense and missense mutations and splice site mutations [3]. A large proportion of mutations presumably result in functional haploinsufficiency for RPS19 by removing transcription from one allele (deletions or insertions) or by nonsense mediated mRNA decay (splice site, non-sense and missense mutations) [4]. A translated RPS19 protein variant can also mediate haploinsufficiency due to reduced stability, inappropriate localization to the nucleoli, reduced affinity to interacting partners and failure to assemble into the pre-ribosome (missense mutations) [5]–[7]. Haploinsufficiency for a ribosomal protein leads to perturbed ribosome subunit synthesis [8]–[10] followed by increased cellular stress, cell cycle arrest and apoptosis [11]. The precise mechanism by which RP mutations mediate the erythroid specific phenotype in DBA is still unclear. It has been hypothesized that erythropoiesis is particularly sensitive to ribosomal protein insufficiency and cellular stress because of a high proliferative and protein synthesis rate [12].

A few gene variants have been described in the non-coding 5′UTR of the *RPS19* gene, i.e. in the first exon upstream of the ATG start codon [13]–[15]. These variants were initially identified in a subset of DBA patients and later in healthy individuals but at a low frequency [14], [15]. In addition, targeted resequencing of the entire *RPS19* gene in DBA patients has revealed a number of non-coding sequence variants and rare polymorphisms localized to introns and flanking sequences [13], [14], [16], [17]. One of the 5′UTR variants (c.-147_-146insGCCA) has been associated with rRNA processing defects but the RPS19 protein levels appeared unchanged in erythroid cells from a patient with this variant [14]. However, it is still unclear if this non-coding sequence variant is transcribed and if it interferes with the translation of *RPS19*. In addition, the distribution of TSS of w.t. *RPS19* have not been carefully analyzed.

The regulation of ribosomal protein expression is critical for cellular adaptation to different requirements. It is well established that the 5′UTR of mRNAs is of importance for gene expression by influencing mRNA stability, subcellular localization, accessibility to the ribosomes and interaction with the translational machinery [18], [19]. Thus, the 5′UTR mediates the adjustment of protein levels to developmental stages, tissue types and growth rate [20]. Conversely, inappropriate expression of 5′UTRs can contribute to abnormal developmental phenotypes and disease [21]–[23]. Furthermore, the 5′UTR of mRNAs encoding ribosomal proteins contains a 5′TOP sequence which enables fast up- or down-regulation of RP levels [24], [25].

The *RPS19* coding sequence and its 5′UTR are highly conserved [7], [13]. However, the significance of the rare polymorphic 5′UTR sequence identified in DBA patients is yet unknown. We examined the transcription of 5′UTR variants and we hypothesized that they affect RPS19 protein synthesis rate as a possible contributing mechanism in DBA. We show herein that the expression of three structural *RPS19* 5′UTR variants leads to a reduced translation into RPS19. Furthermore, RPS19 uses a broad range of TSS with effects on RPS19 translation and with tissue variations.

## Materials and Methods

### *RPS19* expression constructs with different 5′UTR variants

**Figure 1:**
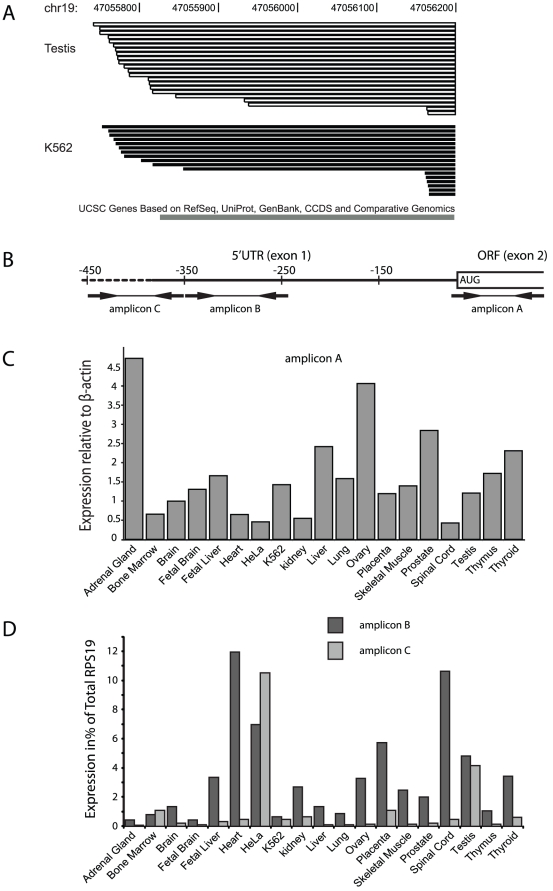
Transcriptional start sites and tissue expression of *RPS19* variants. (**A**) *RPS19* 5′UTR variants in testis and K562 cells. Schematic presentation of 39 different *RPS19* 5′UTRs identified of which 29 are yet undescribed. 5′RACE was performed with 1 µg of total RNA using the GeneRacer® kit (Invitrogen) according to manufacturer's recommendation. The RNA was treated with DNase I to clean samples from genomic DNA. The 5′RACE protocol selected full length G-capped mRNA and ruled out the possibility of partially degraded mRNA. PCR products were cloned into a TOPO-TA vector (Invitrogen) and 122 clones were picked randomly (83 from testis, 39 from K562 cells) and analyzed by bidirectional sequencing. The 5′UTR variants identified are indicated and aligned to the first exon of *RPS19* from databases with a known maximum 5′UTR of 382 nt (bottom). (**B**) A schematic picture of the 5′ region of *RPS19* cDNA (horizontal line) with relative positions of the start codon and the amplicons generated for quantification. Primers used to generate amplicons A, B and C for quantitative PCR are shown as arrows (sequences available upon request). (**C**) Tissue distribution of total *RPS19* as determined by qPCR of amplicon A showing relative expression of *RPS19* normalized to *β-actin* on a panel of primary human tissues and cell lines. Analyses were run in triplicates and the average is shown for each tissue. (**D**) Expression of the amplicons B and C representing longer variants of 5′UTR as determined by qPCR and expressed as a percentage of total *RPS19* expression determined by amplicon A shown in (C).

**Figure 2:**
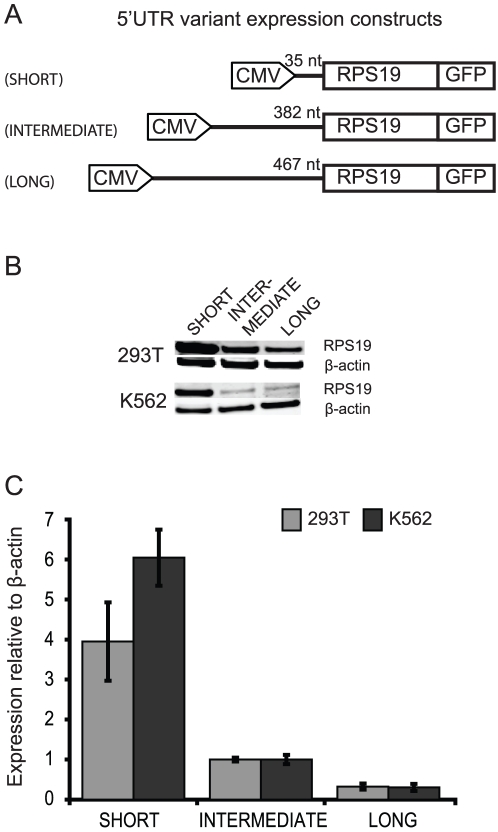
Short *RPS19* 5′UTR translates into more RPS19. (**A**) Sequences corresponding to three w.t. variants of the *RPS19* mRNA including a 35 nt (SHORT), 382 nt (INTERMEDIATE) and 467 nt (LONG) 5′UTR, respectively, were introduced into the fluorescent reporter vector pAcGFP-N1 (Clontech) under the CMV promoter. The expressed fusion proteins consist of a full length RPS19 linked to green fluorescent protein (GFP). (**B–C**) RPS19 protein levels vary with different *RPS19* 5′UTR length. HEK293T and K562 cells were transfected with 5 µg of vector DNA from each of the three 5′UTR variants using Lipofectamine®2000 (Invitrogen). After 48 h, cells were assayed for expression of recombinant protein by fluorescence microscopy and stored at −20°C for further analysis by Western blot (B). (C) Diagram illustrating the relative expression of the three w.t. constructs in HEK293T and K562 cells, respectively. Quantification is based on Western blot analysis in (B) and the expression of RPS19-GFP fusion protein was normalized to β-actin.

*RPS19* cDNA clones with three w.t. variants of the 5′UTR (figure 1) were amplified and cloned into the reporter vector pAcGFP-N1 (Clontech) downstream of the CMV promoter. The resulting constructs encode fusion proteins consisting of a full length RPS19 linked to a fluorescent reporter at the C-terminus (Figure 2A). Three DBA associated 5′UTR variants (c.-147_-146insGCCA, c.-147_-146insAGCC and c.-144_-141delTTTC) were generated by site directed mutagenesis from the pAcGFP-N1-382-S19-5′UTR clone (INTERMEDIATE clone) with Quick change II site directed mutagenesis kit (Stratagene) according to manufacturers recommendations.

### Cell culture and transfection

K562 [26], HeLa [27] and HEK293 [28] cells were cultured in RPMI1640 medium supplemented with 10% fetal calf serum, 2 mM L-glutamine and 20 IU penicillin/streptomycin (all Invitrogen) at 37°C with 5% CO_2_ in a humidified environment. Cells were transfected in 10 cm dishes with 5 µg of the respective vector using Lipofectamine 2000 ® following manufacturer's protocols. Transfected cells were checked for expression of GFP by fluorescence microscopy and harvested using a cell scraper. Cells were collected by centrifugation.

### RNA isolation and quantitative RT/PCR

Total RNA was isolated from K562, HeLa and HEK293 cells using Trizol® reagent (Invitrogen). Quality of RNA was checked using the Agilent RNA 6000 nano kit and the Agilent 2100 bioanalyser according to manufacturer's instructions. RNA samples from a panel of different primary tissues were purchased (Human total RNA master panel #636643; Clontech). cDNA was synthesized with M-MULV reverse transcriptase (MBI Fermentas) using random hexamer primers and 2 µg of total RNA following manufacturer's recommendations. Quantitative real-time PCR was performed in triplicates using platinum SYBR green qPCR supermix UDG (Invitrogen) according to the protocol supplied by the manufacturer. Primer sequences and PCR conditions used to quantify w.t. *RPS19* mRNA with different 5′UTRs are available upon request.

### 5′ Rapid Amplification of cDNA ends (5′ RACE)

5′RACE was performed with 1 µg of total RNA using the GeneRacer® kit (Invitrogen) according to manufacturer's recommendation. Initially, the RNA was treated with DNase I to clean the samples from any genomic DNA. First strand cDNA synthesis was carried out with GeneRacer Oligo(dT) primer and Superscript RT III RACE ready cDNA kit. For amplification of the cDNA end we used the 5′GeneRacer forward primer included in the kit and RPS19 specific reverse primer. The PCR product was cloned into a TOPO-TA vector (Invitrogen) and randomly picked individual clones were sequenced.

### Western blotting

HEK293T and K562 cells were lysed in RIPA buffer supplemented with MG132 proteasome inhibitor (SIGMA), phosphatase inhibitor cocktail 1 (SIGMA), 0.1 mM Sodium vanadate (SIGMA) and protease inhibitor cocktail (SIGMA). Cell lysates were separated on a 10% Bis-Tris SDS-PAGE (NuPage gel; Invitrogen), and transferred to PVDF Immobilon-FL membranes (Millipore). Membranes were hybridized with primary antibodies against GFP (Clontech) and *β*-actin (Abcam). Proteins detected by the antibodies were visualized using Alexa Fluor 680 (α-rabbit) and IRD 800 labeled (α-mouse) secondary antibodies (Molecular probes and LiCor Bioscience, respectively). Western blots were analyzed using the Odyssey® infrared imaging system determining integrated intensities, using *β*-actin as a normalization control as described previously [29].

## Results

### Multiple transcript variants of the *RPS19* gene

The *RPS19* gene spans a genomic region of 11.5 Mb on chromosome 19q and consists of 6 exons [13]. The first exon is untranslated and the start codon is located in the immediate beginning of exon 2. Ribosomal protein S19 exists in one single form consisting of 145 amino acids. Six variants of the *RPS19* transcript have been described with differences only in length of the 5′UTR from the use of alternate transcription initiation sites [7], [13], [30], [31]. This prompted us to search for additional *RPS19* transcript variants and to analyse their effect on RPS19 expression. We performed cDNA 5′RACE with RNA from K562 cells and testis and we determined the transcript sizes and their transcriptional start sites. We identified altogether 31 alternative *RPS19* transcripts with 5′UTRs ranging from 32 to 467 nucleotides. Twenty-nine transcript variants are yet undescribed (figure 1A). The distribution of the *RPS19* 5′UTR length appeared to be different when comparing randomly picked clones from K562 cells and testis, respectively (figure 1). Clones from testis had a distribution towards longer 5′UTRs with the longest clone spanning 467 nucleotides, extending about 100 nucleotides beyond the previously reported longest *RPS19* transcript (Gene bank #BC018616). Longer *RPS19* 5′UTRs appear highly structured as predicted by the RNA secondary structure prediction program Mfold (data not shown).

We then analyzed the relative expression of three groups of *RPS19* transcripts with different 5′UTRs on mRNA from a panel of human tissues (Stratagene) as well as the cell lines K562 and HeLa by quantitative real time PCR. Three specific primer pairs were designed that generate amplicons from the *RPS19* 5′UTR corresponding to nucleotide positions −03 to +89 (amplicon A), −350 to −239 (amplicon B) and −449 to −354 (amplicon C), respectively (figure 1B). Quantification of the RT-PCR products revealed different patterns of 5′UTRs when comparing different tissues and cell lines (1C and D). Transcripts detected by the shortest amplicon (amplicon A) are predominant in all tissues investigated. The expression of longer 5′UTRs defined by the amplicons B and C constitute from <1% to 17% of the total amount of *RPS19* mRNA (figure 1D). No strong correlation was observed between the total amount of *RPS19* mRNA and the relative proportions of longer and short 5′UTR.

### Short *RPS19* 5′UTRs show high translational activity

We made three constructs with *RPS19* 5′UTRs of 35 nucleotides (SHORT; containing the 5′TOP sequence), 382 nucleotides (INTERMEDIATE) and 467 nucleotides (LONG), respectively. Each construct expresses a full-length *RPS19*. The constructs were fused with Green fluorescent protein at the carboxy-terminus (figure 2A) and analyzed when transiently transfected into HEK293T and K562 cells. The SHORT 5′UTR variant is translated 4–6 fold more efficient than the variant with 5′UTRs of 382 nt and >10 fold more efficient than *RPS19* with the 467 nt 5′UTR (figure 2B–C). The results were similar for both cell lines.

### DBA associated 5′UTR variants affect translational activity

**Figure 3:**
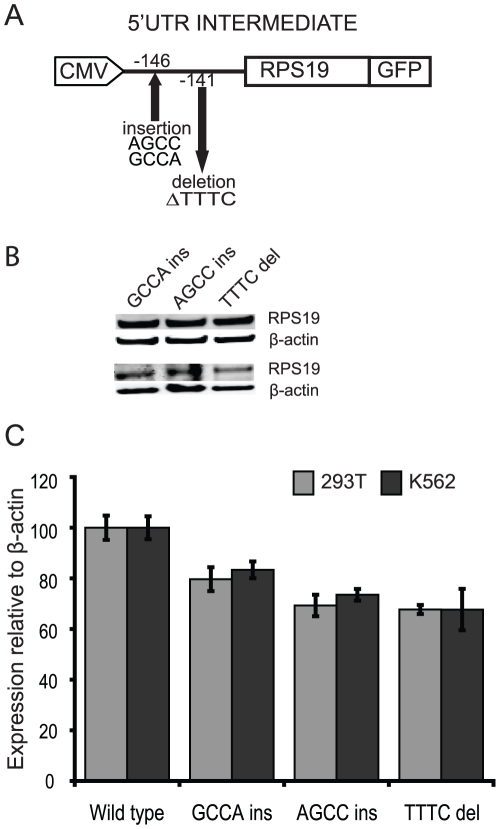
Structural variants in the *RPS19* 5′UTR associated with Diamond-Blackfan anemia affect translational efficiency. (**A**) Three DBA associated 5′UTR variants (c.-147_-146insGCCA, c.-147_-146insAGCC and c.-144_-141delTTTC), were generated by site directed mutagenesis from the pAcGFP-N1-382-S19-5′UTR clone (i.e. the “INTERMEDIATE” w.t. construct of 382 nt) using the Quick change II site directed mutagenesis (Stratagene) kit. (**B**) Western blot of total protein preparations isolated from transfected HEK293T and K562 cells performed as for figure 2B. The constructs express each of the three structural 5′UTR variants. (**C**) Diagram showing the relative levels of RPS19 expressed from the three constructs in 293T and K562 cells, respectively. Quantification was made from Western blot analysis illustrated in (B). The expression from the w.t. “INTERMEDIATE” construct was used as a control and is set to 100.

We next investigated the effect on translation of three distinct polymorphic sequence variants in the 5′UTR of *RPS19* found in a subset of patients with DBA. We introduced two insertions (c.-147_-146insGCCA, c.-147_-146insAGCC) and one deletion (c.-144_-141delTTTC) into the INTERMEDIATE construct (Figure 3A) followed by a transient transfection into K562 and HEK293T cells. The three DBA associated 5′UTR variants reduced the RPS19 levels by approximately 20–30% when compared to the w.t. INTERMEDIATE construct (figure 3B and C). A marked reduction in expression (32%) was observed for the TTTC deletion in both cell lines. The AGCC insertion showed a 30% reduced expression in HEK293 cells and a 26% reduction in K562 cells. The GCCA insertion was associated with a 20% reduction in expression in both cell lines (figure 3B and C). The *RPS19* mRNA levels were similar when comparing cells transfected with the rare variant constructs to cells transfected with the w.t. construct.

## Discussion

The 5′-untranslated region (5′UTR) of an mRNA is an important regulator of translation by influencing e.g. mRNA stability, sub-cellular localization and translational efficiency [32]–[34]. Furthermore, multiple transcriptional start sites and 5′UTRs expressed from a single gene encoding one and the same protein may regulate gene expression through differential expression with respect to developmental stages, tissue type and in response to stimuli [20], [35], [36]. One element that enables fast up- or down-regulation of ribosomal proteins in response to nutrient supply is the 5′TOP sequence, a stretch of 4 to 14 pyrimidines following a Cytidine as the first nucleotide located at the 5′end of an mRNA [37]. This 5′TOP sequence is contained in the SHORT 5′UTR variant used for expression analysis in our study and possibly responsible for fast adaptation of RPS19 levels. The heterogeneous 5′UTRs of mRNAs transcribed from a single gene arise from the use of alternate transcriptional initiation sites and differential RNA processing [38]. It has been estimated that 10–18% of genes express alternate 5′UTRs by multiple promoter usage. Alternate untranslated regions determine tissue specific function and their inappropriate expression can contribute to the development of abnormal phenotypes and disease [39].

We have characterized the *RPS19* 5′UTR variants with respect to expression levels, tissue specificity, and translation efficiency. RPS19 is ubiquitously expressed and mutations in this gene are associated with DBA. The precise molecular mechanisms behind the disease remain unknown but we hypothesized that the expression of different *RPS19* 5′UTR variants may contribute to the regulation of RPS19 protein levels and, ultimately, to DBA. We determined the extent of the *RPS19* mRNA 5′UTR by 5′-RACE on poly(A)+ purified mRNA from testis and K562 cells. The results show an extensive variation in the transcriptional start sites with >30 different 5′UTRs of which 29 are novel. The total amount of *RPS19* mRNA varied considerably between tissues and we observed up to 10-fold differences. Interestingly, bone marrow shows relatively low level of total *RPS19* transcripts when compared to several other primary tissues analyzed. We then investigated the distribution of 5′UTR variants in different tissues. Transcripts were divided into three groups containing a 5′UTR of at least 3 nt, 350 nt and 449 nt, respectively. Our data indicate clear differences in the distribution of *RPS19* 5′UTRs when comparing different tissues. The amplicon corresponding to the shorter 5′UTR (amplicon A) constituted between 83% to >99% of *RPS19* transcripts but without correlation to the variation in total amounts of *RPS19* mRNA.

To get a better insight into the translational regulation of *RPS19* 5′UTR variants we investigated the RPS19 levels expressed from constructs with three distinct 5′UTRs length. The SHORT 5′UTR variant, spanning a 35 bp 5′UTR, is translated four to ten fold more efficiently than the two longer variants with 5′UTRs of 382 bp and 467 bp, respectively. This is also consistent with the analysis of variable 5′UTR length of other genes [34], [36]. A possible explanation is that the SHORT variant exhibits a less complex secondary structure, facilitating scanning by the translation machinery. The reduced translation from transcripts with longer 5′UTRs may be related to the more complex secondary structures, making the transcripts less accessible for translation. The functional significance of the longer *RPS19* 5′UTRs is unclear, but may be of importance for 5′TOP independent translation providing baseline amounts of RPS19. The shorter variants could in this case be used for a fast adaptation of RPS19 levels in response to cellular needs. In combination, our observations suggest a large variation in *RPS19* mRNA levels between tissues as well as in TSS used. The predominant and shorter 5′UTRs are more efficiently translated and may directly reflect the levels of RPS19.

We then analyzed the effect on translation of rare sequence variants in the *RPS19* 5′UTR found in subsets of DBA patients. We confirmed that the polymorphic 5′UTR variants are indeed transcribed and we hypothesized that these transcripts affect translational efficiency. We therefore investigated the RPS19 levels expressed from constructs with each of the two insertions (c.-147_-146insGCCA, c.-147_-146insAGCC) or the deletion (c.-144_-141delTTTC), respectively. All three variants result in significantly reduced RPS19 levels when compared to the corresponding wild type sequence. A possible explanation is that the mutation causes the mRNA to adopt a more complex secondary structure that represses translation. It is noteworthy that the observed reduction in RPS19 levels in our cell-systems is related to a relatively large proportion of the “INTERMEDIATE” *RPS19* transcripts containing each of the specific 5′UTR variants. Still, the observed effects of the three 5′UTR variants associated with DBA do not result in haploinsufficiency and, accordingly, the impact on RPS19 levels *in vivo* would depend on the relative amounts of longer *RPS19* mRNAs. Although these 5′UTR variants may lead to suboptimal conditions for growth and differentiation of tissues sensitive to reduced RPS19 levels it is likely that additional factors are required for overt clinical forms of DBA. Our results are consistent with the increased ratio of 21S/18S pre-rRNAs associated with the c.-147_-146insGCCA variant observed previously [14]. Failure to detect reduced RPS19 levels in that study may be due to low abundance of longer transcripts and/or minor changes in RPS19 levels in the cells analyzed.

Our combined findings suggest complex regulatory mechanisms of RPS19. *RPS19* uses a broad range of TSS with tissue specific differences and shorter 5′UTRs are more efficiently translated. We also show that DBA associated 5′UTR variants of *RPS19* are less efficiently translated. Further investigations are now required to understand how RPS19 is regulated in different tissues both at the transcriptional and the translational level. These studies may clarify the distribution and levels of *RPS19* 5′UTR variants as well as RPS19 protein levels at different stages of erythropoiesis. Thus, analysis of the *RPS19* TSS used in erythroid precursor cells may provide valuable information in search for molecular mechanisms behind DBA.
